# Electric Heat in Dental Practice

**Published:** 1896-01

**Authors:** Levitt E. Custer

**Affiliations:** Dayton, Ohio


### International Dental Journal.

Vol. XVII. January, 1896. No. 1.

### Original Communications.[1]

### ELECTRIC HEAT IN DENTAL PRACTICE.[2]

BY LEVITT E. CUSTER, B.S., D.D.S., DAYTON, OHIO.

In the ordinary applications of heat, the gas and carbon products, while unpleasant and difficult to control, are not injurious; but in a few of the arts and some of the processes of dental practice the presence of these products makes it a serious matter. On this account we are glad to avail ourselves of a form of heat not new, but which has become practical only since the introduction of commercial electricity.

Electric heat differs from all other forms of heat in that it is not itself a process of combustion, neither is it a chemical action, nor one of friction. It does not consume and does not emit. But, apparently without any cause, the conductor of the current rises in temperature and radiates this pure heat of which we avail ourselves.

There are three terms in electricity which represent its very foundation,—the *ampère*, *volt*, and *watt*. These respectively represent the quantity, pressure, and power, and, although frequently used synonymously, are widely different. The ampère is the

[1] The editor and publishers are not responsible for the views of authors of papers published in this department, nor for any claim to novelty, or otherwise, that may be made by them. No papers will be received for this department that have appeared in any other journal published in the country.

[2] Read before the Academy of Stomatology, Philadelphia, October 15, 1895.

measure, the volume of current, or, we may say, the active principle of electricity, the working agent. It is that which produces heat, electrolysis, and magnetism. We may say it is electricity itself, and we speak of it as a quantity of so many ampères.

The ampère is of no value, however, unless it is in motion, and the measure of that force which puts it in motion is the volt. While all so called conductors of electricity allow electricity to flow through them, they at the same time offer more or less resistance to its flow. To overcome this resistance there must be some force from behind, and this has been termed the volt. For this reason we speak of a current at so many volts pressure. The incandescent lamp glows because the ampère of current is flowing through its filament; but at the power station the dynamo is forcing the ampère in at one hundred and ten volts pressure, enough to carry it to the lamp and through its filament.

The watt is the term for power, and represents electric energy. It is the aggregate, or summing up, in one word, of the output or consumption of electric energy. It is the product of the ampères multiplied by the volts, and is readily converted into what we call horse-power by considering seven hundred and forty-six watts to be equal to one horse-power. It does not matter how the watts are made up, whether more of volts or of ampères; so long as their product equals seven hundred and forty-six it is one horse-power. A current of seventy-five ampères flowing under a pressure of ten volts will do work equal to about one horse-power, or a current of one ampère at seven hundred and forty-six volts will produce one horse-power. A sixteen-candle-power lamp consumes fifty-five watts; at one hundred and ten volts that would be half an ampère each, and thirteen such burning at one time represents an expenditure of one horse-power.

The heating element of electricity is the quantity or ampères. Volts do not heat; they only force the quantity through the conductor. If it requires six ampères of current at two volts to heat a cautery loop an inch in length, the same quantity of current at one hundred and ten volts would heat a piece of the same wire fifty-five inches in length. You will recall an electric exhibit at the World's Fair in which a bar of iron an inch in thickness was heated to redness in a few minutes. This was done by forcing through possibly a thousand ampères of current at a few volts pressure. Such a current is not dangerous to life, because the body offers so great a resistance that the few volts cannot force the ampères through.

Besides the quantity, or ampères, electric heat also depends upon another factor. There can be no perceptible heat unless the current meets with resistance to its flow. As the quantity is increased the heating power is increased, but this power is not apparent until the current meets with some resistance. The unobstructed flow of any quantity of the fluid does not produce heat. It is only when a poor conductor of electricity is placed in the circuit that we have this manifestation.

Electric resistance is of two kinds,—the resistance of the material of which the conductor is composed, and the cross-section of the conductor. All metals are comparatively good conductors of electricity, yet these vary in their conducting property. Copper stands at one extreme and German silver at the other. In the electric gold annealer and porcelain oven platino-iridium wire is used, because it is a poor conductor and does not oxidize or readily melt.

The size of the wire is the other factor in the determination of the heat. With a given length of wire the resistance increases as the diameter of the wire decreases,—that is, a small wire has less carrying capacity than a large one, so that when the same amount of current that is easily conducted by a large wire is forced through a small one, the condensation, I will term it, produces heat; so we would say that, with the same quantity of electricity at a given pressure, heat is produced according to the resistance of the conducting agent.

The atmosphere is practically a non-conductor of electricity, yet under certain circumstances it does allow the passage of an electric current. When a current has been established by bringing two terminals together, the electricity continues to flow, even after the ends have been separated. It leaps the intervening thi[illegible]ess of air in the form of the voltaic arc, and continues until the distance becomes too great. This action develops so much heat that it has led to the supposition that electricity is another form of heat. When the oppositely electrical clouds unite it passes through the atmosphere as lightning; when the Leyden jar discharges it is the spark; or when it lights the street-corner it is the arc. The heat of the arc is so intense that by our present methods it cannot be measured, but is estimated at 6000° F. With the ordinary Edison current I find that we can not only melt platinum and iridium, but in small quantities we can vaporize them.

Electric heat, when obtained by heating a conductor that does not oxidize, differs from other forms of heat in that it is without gas, noise, or odor, and on that account is of special value in dental practice. Electric heat also differs from that obtained from other sources in that it can be controlled and regulated with the utmost precision. A given number of ampères, forced through a known conductor at a given pressure, always produces the same amount of heat, and it is possible even to calculate beforehand precisely how much heat will be developed by the different arrangements of volts, ampères, and resistance.

No case in dental practice, or in any other practice, for that matter, calls for a blast of air exactly at blood temperature so much as an almost exposed pulp, and no instrument so nearly meets this requirement as the electric warm-air blast. With air at a constant pressure, which is carried over electrically-heated wires at a constant heat, the air escapes from the syringe-nozzle at a uniform temperature. The heat can be varied by the operator in three ways,—by manipulating the air-pressure, by altering the electric heat by means of the rheostat, or by varying the distance of the syringe-point from the cavity. After a little experience the operator can dry out the cavity without the slightest pain to the patient, and, if the air has passed through a wash-bottle of alcohol or any such agent, it carries its vapor with it.

The cautery and electric root-dryer are both familiar to you, and are examples, on a small scale, of the heating power of electricity. These instruments can both be successfully operated on the Edison current by throwing in about ten ohms resistance and taking off the cautery heat by what is known as a "shunt" current.

The value of any heat for sterilization is duly recognized, and for some time I have been satisfactorily using the electric oven, raised not quite to the heat of withdrawing the temper of the instrument. The heat may be maintained all day long, and the cleanliness and simplicity recommends it. Gutta-percha is softened with accuracy when placed on a soapstone slab resting on the oven, and the waste heat rising from the whole appliance is utilized for keeping water warm for the syringe.

There are two processes in dental practice which call for absolute purity and uniformity of heat. Upon recognizing the special fitness of electricity for meeting these conditions, I some years ago devised an appliance for annealing gold thereby, and one more recently for fusing porcelain.

The heat produced by electrically heating a mat of coiled platinum wire is the cleanest, most uniform, and most accurately controlled of all forms of heat. The cohesive property of pure gold is supposed to be developed by heating to such a temperature as to drive off the gases condensed upon its surface, the principal of which is ammonia. The alcohol and Bunsen flame are ordinarily used for this purpose; but who is certain that better results may not be obtained by subjecting to a heat free from the products of combustion, as well as to the danger of smoking and the exposure to unconsumed gases? The electric annealer effectually overcomes these dangers. The heat, being derived from electrically-heated platinum, itself a noble metal, is absolutely free from gas of any kind. The heat is radiated from a mat of platinum coils, and is quite uniform at all parts, so that the gold is not only thoroughly annealed, but it is evenly annealed. It is impossible to evenly heat a piece of gold, held with a pair of pliers, over a flame of any kind. The thin edges of the gold will be fused, while the part between the pliers will be scarcely warm. The accuracy with which electric heat can be controlled also recommends its use for annealing. By means of the rheostat any degree of heat to the melting-point of platinum may be obtained. From my experience with the electric annealer, however, I find that cohesion is developed at a much lower degree than at first supposed. It is never necessary to heat even to redness, let alone fusion. The heat may be so low that the gold may be subjected to it for hours, or for days, even, without any injury, and still be highly cohesive.

Electrically annealing gold saves time in many ways. The gold requires no attention, is ready for use at all times, and, the heat not being high enough to take the temper from the plugger-point, this instrument may be used to pick up the gold, thus saving the time of changing instruments. After six years' use, I am free to say that next in usefulness to the dental engine is the electric gold annealer.

The latest practical application of electric heat in dental practice is the electric oven for fusing porcelain. From the time of Allen and Hunter, or from the very beginning of porcelain work, the question of heat has been a serious one, and the principal reason that continuous gum has not been more popular is the difficulty and uncertainty in the production of heat. The heating principle of the oven is an electrically-heated platino-iridium wire, or the gold annealer in the form of an oven. In using this new source of heat I departed from the old muffle-shaped oven to one more in keeping with this new agent. It consists of an upper and lower section, flask-shaped, with an inner cavity amply large enough to contain a set of teeth, and of such form and arrangement of the wires that all parts receive the same degree of heat. Upon the whole inner surface is embedded the electric conductor, just deep enough to be supported while so highly heated, and yet to radiate its heat directly into the oven cavity. The upper half is hinged to the lower, which automatically makes the electric connection upon being closed. There are two openings through which the fusing process may be watched. These are placed at such positions that rays of light entering one will be reflected out by the plate through the other. This overcomes the intense glare of the heat, and at the same time brings the plate clearly into view, making it possible for an inexperienced operator to accurately determine the degree of fusion.

There are many other advantages offered by the electric oven.

The source of heat being a noble metal electrically heated, it will be readily seen that a heat is obtained that is unlike any heretofore used for this purpose; and since it gives rise to no products of combustion, it is an impossibility to produce what is known as "gasing," and porcelain fused by this method not only possesses unusual clearness, but appears to be more dense.

The accuracy with which electric heat can be regulated by means of the rheostat, the cleanliness and simplicity, freedom from noise and odor, are advantages over the older forms. With electric heat we have something definite. It is not affected by bad coke, bad oil, gas, or unequal draught. A given number of ampères forced through a conductor at a given pressure produces a heat as definite and results as positive as a rule in mathematics.

We can control electricity, and the opportunity is now open for any number of automatic appliances to regulate the heat according to the porcelain treated. I have up to this time devised a clock attachment to the rheostat whereby the current is gradually raised and cut off at a set time; second, a fusible button of porcelain placed in the oven at the time of fusing, which rings a bell when the porcelain fuses; third, an electric thermometer, whereby the temperature of the oven is quite accurately told by the rise of mercury in the tube; and, fourth, an ammeter, the swing of whose arm is in proportion to the heat of the oven.

These appliances are mechanical and quite accurate; but it is such a pleasure to sit down and observe the different stages of fusing that the experienced operator who has had to handle his case at a distance and with so much anxiety takes delight in watching and varying the heat at will, as he would play with a toy.

The cost of operation is very small indeed. The oven, as now made, consumes six ampères, which would be about two cents per fuse of thirty minutes.

In case a wire should burn out by accident, this break in the current automatically cuts the current off, so that no further damage is done, and it requires but a few minutes to repair the break in a way that is as good as new.

While the electric oven operates best when used on the Edison current, it is still very satisfactory on the fifty-two-volt alternating, the two-hundred-and-twenty-volt or the five-hundred-volt currents.

In the practical operation of the oven the procedure is very simple. The case is placed on the tray in the lower section, and the upper is then closed down. The lever of the rheostat is placed on the first button, and heat for thoroughly drying out the case is quickly obtained. When the operator is satisfied that there is no more moisture present, he begins raising the heat by pushing the lever to the right. If he allows two minutes to each button, it will require from twenty to twenty-five minutes to reach the fusing point. If it is a crown or a bridge, less time may be consumed in raising the heat without danger to the case, and it may be fused in from ten to fifteen minutes by throwing the lever over more rapidly. In practice I do not even measure off the time to each button, but fuse while I am operating. From time to time, as it occurs to me, I throw on two or three buttons at a time, according as the interval has been, until I have reached the third from the last button, on which it is allowed to remain until I have three minutes in which to give it my undivided attention. The porcelain is just ready to drop into a fuse, and upon throwing on the last button the successive stages and degrees of fusion are clearly made out.

In the first stage the porcelain is still in the powder form, and appears like snow. Presently it begins to drop into a fuse, and the snow-like appearance changes into a dead, indistinguishable mass; the particles are now beginning to coalesce. Gradually the surface, with all its inequalities, comes clearly into view, and presents a glistening appearance; continuing the heat a moment longer, the porcelain becomes more liquid, and the inequalities of the surface assume a more even appearance. Since the eye can be brought so close to the plate with the electric oven, and since the plate is brought clearly to view by the arrangement of the two sight openings, the operator is not guessing by the quantity of heat or the general appearance of the plate, but he is actually observing the different particles of the porcelain itself, and for that reason the fineness of his fusing is always assured and at his perfect control.

When the desired heat has been obtained the lever of the rheostat is thrown back, which cuts the current off. At that very instant the heat begins to go down, so that there is neither overfusing nor loss of brilliancy in the gum color. If it is the first or second baking of the case, the stoppers need not be inserted, and the case can be taken out in a short time; but if it is the last fusing, after a few moments' time has elapsed and the case has become a dull red, the stoppers should be inserted and the case allowed to gradually cool.

It does not matter how nearly I have approached the ideal the prosthetic dentist has long sought in the invention and perfection of this appliance, there will be some who will find objection in some particular. It is but just to mention, however, that the objections have all come from persons who have not examined into the practical working of the instrument, but have stood afar off with forbidding gestures.

A prominent porcelain worker says it will be impossible to fuse thick portions, as well as thin, in such an oven. To that objection I would answer that in the old form of muffle it would be possible to do that; but in an oven which radiates its heat equally from all directions, as the electric oven does, the reasonable thinker will acknowledge that the thick would be fused equally as well as the thin, especially when we bear in mind that the porcelain is in the form of a powder, light and loose, before it fuses, so that the thicker portions are permeated by the heat as well as the thin, and practical tests prove this statement to be correct.

The older practitioners, who have been accustomed to working with large ovens which retain their heat for a long time, insist that the case must be taken out and put in an annealing oven. To those persons I would say it can cool in the oven just as well; not only that, but the case is not subjected to a sudden change of temperature, as must certainly occur when removing to the annealing oven; and on that account we claim it is rather an advantage to have the case remain in, and all cool down together. To those who think they must still do this, however, I would say they can easily do so until they find it unnecessary.

There is a common objection that it is an electrical instrument, and, like many such appliances, is liable to get out of order, or that it requires an electrician to operate it. To that objection I can only say that it is much less complicated than a motor, and any one who is reasonable can operate it. On account of the special fitness of electricity in dental practice, it is becoming late in the day for any person to acknowledge his inability to take up the common applications of electricity, or to forbid their adaptation in his practice on general principles. He should bear in mind that we are not to-day dealing with the uncertain batteries of the past, but with the certain results of that Llewellyn genius, Edison.

It would seem that electricity has given us all we could ask for; and yet I am forced to say that the properties and applications of electricity are just unfolding, and the demands of dental practice of the future will keep pace with, if not in the lead of, electric progress. It is not a dream when I say the time is coming when electricity will have its place on the dental curriculum as much so as materia medica or metallurgy has now. No profession, science, or art has such varied demands in its practice as dentistry, and no single agent more nearly meets these than electricity.

